# Feasibility of diffusion-weighted imaging with DWIBS in staging Hodgkin lymphoma in pediatric patients: comparison with PET/CT

**DOI:** 10.1007/s10334-018-0726-4

**Published:** 2018-11-29

**Authors:** Dobromila Baranska, Katarzyna Matera, Michal Podgorski, Magdalena Gorska-Chrzastek, Karolina Krajewska, Joanna Trelinska, Piotr Grzelak

**Affiliations:** 1 Department of Diagnostic Imaging, Polish Mother’s Memorial Hospital-Research Institute in Lodz, Rzgowska 281/289, 93-338 Lodz, Poland; 2 PET/CT Laboratory, NZOZ MCD Voxel, Polnocna 42, 91-425 Lodz, Poland; 3 Department of Pediatrics, Oncology, Hematology and Diabetology Medical, University of Lodz, Pankiewicza 16, 91-738 Lodz, Poland

**Keywords:** Apparent diffusion coefficient, Diffusion-weighted magnetic resonance imaging, Hodgkin lymphoma, Positron emission tomography/computed tomography, Standard uptake value

## Abstract

**Objective:**

The aim of the study was to evaluate feasibility of diffusion-weighted whole-body imaging with background body signal suppression (DWIBS) method in diagnosing Hodgkin lymphoma in pediatric patients and to compare it with 18F-FDG PET/CT as a gold standard.

**Materials and methods:**

Eleven patients (median age 14) with newly diagnosed Hodgkin lymphoma were examined with 18F-FDG PET/CT and MRI including whole-body DWIBS sequence (*b* = 0, 800 s/mm^2^), before the oncologic treatment. About 26 locations of lymphatic tissues were evaluated visually and quantitatively using ADC_mean_ (DWIBS) and SUV_max_ (18F-FDG PET/CT), respectively.

**Results:**

All affected lymph node regions (*n* = 134) diagnosed in 18F-FDG PET/CT were found with DWIBS, presenting decreased diffusion. Significant correlation was found between ADC and SUV values (*R*^2^ = − 0.37; *p* = 0.0001). Nevertheless, additional 33 regions were recognized only by DWIBS. They were significantly smaller than regions diagnosed by both methods.

**Discussion:**

Agreement between DWIBS and 18F-FDG PET/CT for detection and staging of malignant lymphoma is high. DWIBS can be used for the evaluation of pediatric Hodgkin lymphoma.

## Introduction

Hodgkin lymphoma (HL) incidence for children aged 15–19 had been reported as 15.9 per million. It constitutes the third most common malignancies in this age group [1]. There are several diagnostic options for the initial staging and restaging of the Hodgkin lymphoma. According to latest European recommendations (EuroNet-Pediatric Hodgkin’s Lymphoma Group-C2, EuroNet-PHL-C2), the whole-body PET/MR (Positron Emission Tomography–Magnetic Resonance), both method combined with chest CT (Computed Tomography) and abdominal ultrasound are recommended. However, due to low availability, more commonly, whole-body 18F-FDG PET/CT (2-[Fluorine-18]-fluoro-2-deoxy-d-glucose positron emission tomography/computed tomography) is used [2–4]. This examination was a gold standard method in former recommendations (EuroNet-PHL-C1/Interimphase/LP1) and characterized by sensitivity and specificity for recognition of nodal disease reaching 87.5% and 85.6%, respectively [5–7].

As far as the safety is concerned, in PET, the amount of radio-labeled pharmaceutical is extremely small (0.01–10 µg), having essentially no pharmacological effect. However, during single 18F-FDG PET/CT examination, patient receives about 8.8 ± 1.8 mSv. Moreover, according to actual guidelines, this examination is repeated in most cases 2 or 3 times a year (in the first year of disease), and then performed in all cases of suspected recurrence of the disease [8, 9]. Even though, the CT study correlated with 18F-FDG PET has been highly optimized and is considered as a low-dose technique [10, 11]. Contrary to this procedure, an addition of the CT examination as a separated study (e.g., chest scan) considerably increases the dose of radiation [12]. Thus, high cumulative effective dose (reaching 60–113 mSv) carries a risk of long-term radiation complications, especially for pediatric patients [13, 14].

The limitation of the 18F-FDG PET/CT technique is its moderate specificity, with many false-positive foci in the intestinal region associated with benign conditions such as reactive lymphatic hyperplasia, inflammation or red cell senescence [15]. With more children surviving lymphomas, finding safer and less toxic diagnostic options is a must to reduce the risk of late radiation complications such as secondary cancer and inheritable DNA mutations [16, 17].

Diffusion-weighted whole-body imaging with background body signal suppression (DWIBS) may be a good, radiation-free alternative [18, 19]. This method is based on the measurements of Brownian motion of water molecules in biological tissue. In many pathological conditions, water diffusivity is impeded (low) due to, e.g., increased neoplastic cellularity or swelling in inflammatory or infectious lesions. It also concerns lymphomas, where cells are densely packed and randomly organized, inhibiting an effective motion of extracellular water [20–23]. DWIBS provides cross-sectional imaging of the entire body, with a high soft-tissue contrast, and functional information [24]. It is already employed in the follow-ups of patients with lymphoma [25–27]. It is included in EuroNet-PHL-Interimphase trial, which is still being continued by some European countries. Due to high spatial resolution, this technique allows for evaluation of the nodal space even in children, and thus accurately determining lymphoma involvement [28, 29].

There are still little data on the accuracy of DWIBS technique in initial staging of Hodgkin lymphoma in pediatric patients [19, 20, 28, 30, 31]. Thus, this study aims to compare the accuracy of lymphatic regions recognition between DWIBS technique and the 18F-FDG PET/CT as a gold standard in a group of pediatric patients newly diagnosed with Hodgkin lymphoma.

## Materials and methods

### Patients

In 11 patients newly diagnosed with Hodgkin lymphoma (8 girls and 3 boys, median of age 14, range 8–16 years), who followed a standard diagnostic EuroNET protocol with 18F-FDG PET/CT examination, we performed DWIBS study in short time intervals (3 days). Newly diagnosed, histologically proven lymphoma and age below 18 years were inclusion criteria. Besides, each patient with a stage > IIA underwent bone marrow biopsy to determine the bone marrow involvement. All visible groups of lymphatic nodes were included in the analysis (with the exclusion of extra-nodal lesions). The study protocol was approved by a local bioethical committee (decision number 65/2017) and was in accordance with the Declaration from Helsinki. Informed consent was obtained from all the individual participants included in the study.

## 18F-FDG PET/CT procedure

18F-FDG PET/CT procedure was performed using a PET/CT (*Discovery iQ 4*-*Ring, General Electric Healthcare Milwaukee, WI, USA*) scanner. Images were acquired in caudal–cranial direction from the proximal one-third of the thigh to the skull base.

After at least 6 h of fasting (the serum glucose level was < 130 mg/dl in all patients [32]) and 60–65 min before PET imaging, each patient was injected with 3 mL of the 18F-FDG solution (2.5 MBq/kg of body weight). CT was performed immediately before PET with the patient in the same position. PET was 3 min per bed position (about 20 cm in length). The total examination time was about 10 min. The FDG uptake can be evaluated semiquantitatively using the standardized uptake value (SUV). The SUV is the activity in the lesion in MBq/mL corrected for the weight of the patient and the dose of administered FDG.

PET, CT, and fused 18F-FDG PET/CT images were analyzed on a workstation (*Intellispace Portal Workstation v7.03, Philips Healthcare Nederland*) to evaluate the maximum SUV (SUV_max_) [33]. 3D ROI was placed over each lesion. Malignancy staging and delineation were performed by visual assessment of MIP images, multiplanar views (axial, coronal and sagittal) of PET, and fused 18F-FDG PET/CT images [34]. The lesion’s SUV_max_ with each visible 18F-FDG uptake, among all foci was identified [35]. This can be achieved by satisfied spatial resolution, which in IQ-type scanners is approached by a specific detectors structure. In this apparatus model, the spatial resolution could range from 4.2 mm at 1 cm to 8.5 mm at 20 cm [36]. Analysis process was determined by reconstruction algorithms (*View Point HD* with point spread function modeling and *Q.Clear*). This *Q.Clear* reconstruction improves the PET image quality, with higher recovery coefficients and lower background variability [37, 38].

For the purpose of correlation between the Whole-Body (WB) MRI and 18F-FDG PET/CT, a lesion-by-lesion analysis was performed. SUV_max_ was recorded from the largest uptake area visible in PET. Contouring was performed by radiologist with 10 years of experience in reporting PET/CT. The lowest mean apparent diffusion coefficient (ADC_mean_) of ROI was generated from the ADC map.

### Diffusion-type WB MRI technique for staging Hodgkin lymphoma

All examinations were performed using a 1.5-T MRI system (*Ingenia Omega HP, Philips Healthcare Nederland*) equipped with a torso coil (*dStream Torso*, 32 channels, *Philips Healthcare Nederland*), head and neck coil (*dStream HeadNeck*, 16 channels, *Philips Healthcare Nederland)* to cover head, neck and trunk [38]. Two age-dependent protocols were prepared for patients younger than 10 years and older than 11 years. The protocol was (or The protocols were) split over (a/the) field of view covering patient’s body from skull base to mid thighs (as designed in EuroNet-PHL protocols). WB MRI exam consisted of coronal T1-weighted turbo spin-echo (T1W_TSE) sequence with breath-holding in chest and abdomen, coronal fat-suppressed T2-weighted (T2W_STIR) short tau inversion recovery (STIR) with respiratory triggering in chest and abdomen. Diffusion-type WB MRI was performed with DWIBS using echo planar imaging (EPI) during free breathing [4]. Each listed sequence was equipped with parallel acquisition technique (sensitivity encoding, SENSE), which is responsible for reaching an increased spatial resolution and decrease acquisition time. Thus, application of SENSE makes WB MRI examination an adjusted tool for reaching an excellent soft-tissue contrast [5, 39, 40]. Double *b* values were selected for ADC calculation, 0 and 800 [41]. Coronal maximum intensity projection (MIP) images were reconstructed from STIR and DWIBS separately. The MIP thickness was displayed and calculated automatically from the patient’s axial or coronal field of view. In addition, an axial turbo spin-echo (T1W_TSE) scan was also included for further characterization of suspected lesions. The examinations were performed without contrast agent administration. Total examination time was estimated for about 35 min. Table 1 shows the sequences used in protocol.

**Table 1:** WB MRI sequence protocol parameters

Parameter	T2W_TSE	DWIBS	T1W_TSE	T2W_STIR
Echo	SE fast imaging mode TSE Multishot	IR fast imaging mode EPI Single-shot double b-factors	IR fast imaging mode EPI Multishot	IR fast imaging mode TSE Single-shot
TR (ms)	12021/13023*	6260/6335*	11281/11986*	5.4
TE (ms)	100	65/67*	70	1.72/3.6
Flip angle (°)	90	No	No	15
NSA	2	4	1	1
Slice thickness (mm)	3	5	5	2.2
Slice gap (mm)	0.8	1	0.7	0
WFS	Maximum	Minimum	Maximum	Default
SENSE	Yes	Yes	Yes	Yes
Max b factor	No	800	No	No
Breath hold	No	No	Expiration	Expiration
Phase encoding	RL	AP	RL	RL
Estimated time (mm:ss)	04:13/05:00*	03:02/03:04*	05:24	00:34/00:36*
Number of sequence used	2 × (chest, head and neck)	4 × (head, neck, chest, abdomen)	1 × chest	4 × (head, neck, chest, abdomen)

Patient age: up to 10

* Above 11 years old

### Evaluation of data correlation

It should be initially noted that there is no generally valid WB MRI protocol; thus, for the study, the original total body protocol was created. However, until now, different combinations have been used to formulate the most matched protocol for lymphomas [28, 42]. In our study, WB MRI evaluation began by reviewing the MobiView of the T2W_STIR, followed by the correlation with the axial DWIBS and axial morphologic images.

In lymph nodes evaluation, we assimilated EuroNet-PHL recommendations for CT examination. Thus, lymph nodes were recognized in the T2W_STIR sequence as positive (lymphomatous) when any diameter was > 2 cm, suspicious when it ranged between 1 and 2 cm, and negative if it was < 1 cm. Additionally, in this study, a lymph node was suspected of malignancy if it showed a focal signal intensity equal to or greater than the organ with usually highest diffusion signal intensity in the same region (brain, salivary glands, tonsils, spleen, gallbladder, adrenal glands, prostate, spinal cord, peripheral nerves, bone marrow and reproductive organs) despite the size [18, 42]. Lymph nodes were not measured on diffusion-weighted images because the measurements are highly dependent on the applied window level and window width. Instead, diffusion-weighted images were used only to detect potential nodal abnormalities and to estimate ADC values. Also, any pathological signals from DWIBS had to be confirmed by morphological scans.

After confirming the node size in the STIR image, a free-hand region of interest (ROI) measurement with ellipsoidal contour was then drawn on DWIBS (b800) image to encompass the entire cross section of the lesion. ADC of the lymph node was performed on the ADC map, automatically. Each visible lymph node was measured on one slice, on which it appeared to be the largest. Mean ADC and ellipse surface area of each lesion were assessed. The high coverage achieved by DWIBS allows to classify disease to one of the IV spreading stages [30]. We used Ann Arbor scale to classify the lymphoma and refer it to the staging performed based on 18F-FDG PET/CT.

### Statistical analysis

In statistical analysis, we had two goals. Firstly, to assess whether the DWIBS examination allowed to recognize affected regions with the same accuracy as the 18F-FDG PET/CT. For analysis, we used the Chi square test. The Mann–Whitney test was used to compare size of lesions between two groups. Secondly, to evaluate the association between the SUV_max_ the ADC_mean_, particularly/mainly, to check whether the ADC can be used as a quantitative surrogate of neoplastic process aggravation. We evaluated normality of data distribution with the Shapiro–Wilk test and the due to not-normally distributed variables, we applied Spearman’s rank correlation test. In addition, this test enabled to evaluate a correlation between an area of the lymph node and ADC_mean_. The statistical analysis was performed using Statistica 12 software (*StatSoft Polska, Cracow, Poland*). A *p* value below 0.05 was considered significant. The results are presented as a mean and standard deviation.

## Results

**Fig. 1:**
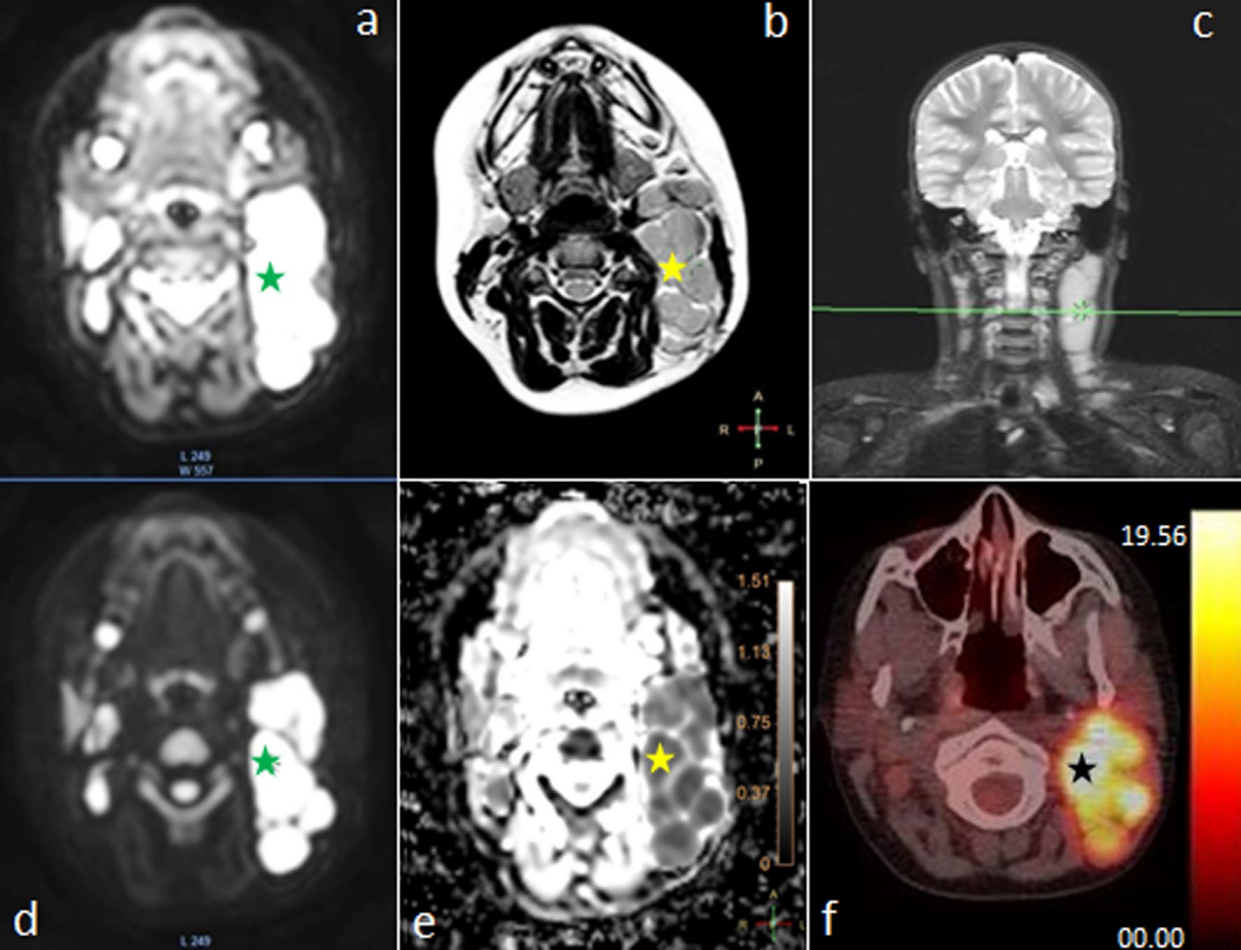
Images of a 14-year-old girl with Hodgkin lymphoma, showing the same enlarged cervical group of lymph nodes: **a** WB MRI, axial of b0 DWIBS image hyperintensity restricted diffusion (green star), **b** WB MRI, axial of T2-weighted image with isointensity region (yellow star); **c** WB MRI, coronal MIP reconstruction of head and neck STIR image with high signal intensity region (little green cross on green, horizontal line); **d** WB MRI, axial of b800 DWIBS image subtraction with restricted diffusion region (green star); **e** WB MRI, axial of ADC map from DWIBS images subtraction with hypointensity restricted diffusion region (yellow star) and ADC scale; **f** 18F-FDG PET/CT axial image showing 18F-FDG uptake (black star) of cervical region and SUV scale

**Fig. 2:**
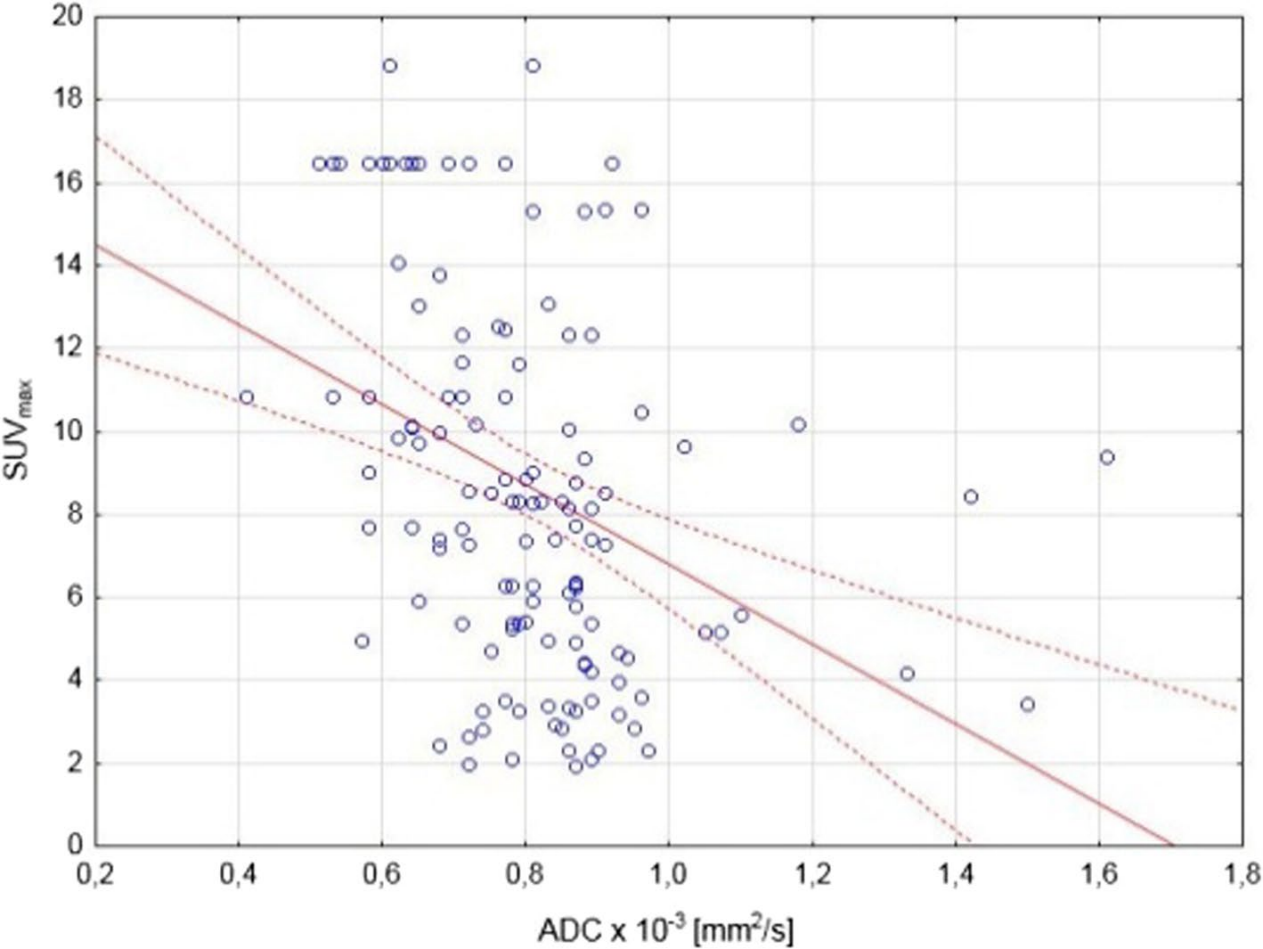
Correlations between ADC_mean_ and SUV_max_ variables

All patients were diagnosed with multiple locations of neoplastic process (median number of 16 locations, range 7–26). Lesions with pathologic features in both imaging methods were termed as recognized regions. The lesion’s SUV_max_ with each visible 18F-FDG uptake, among all foci was identified. For correlation between apparent diffusion coefficient (ADC) and 18F-FDG quantification, a lesion-by-lesion analysis was performed. In this analysis, SUV_max_ was recorded for the lesion with the largest diameter and the lowest mean apparent diffusion coefficient (ADC_mean_) value on the WB MRI (Fig. 1). Due to a better spatial resolution of MRI examination comparing to the 18F-FDG PET/CT, it was possible to delineate nodal groups better and qualify into anatomical regions properly, for instance, not to mediastinal but to axillar or subclavicular region. All regions of increased metabolism visible in 18F-FDG PET/CT presented decreased diffusion in DWIBS. Moreover, values of ADC_mean_ and SUV_max_ correlated significantly with each other (*R*^2^ = − 0.36; *p* = 0.0002) (Fig. 2).

**Fig. 3:**
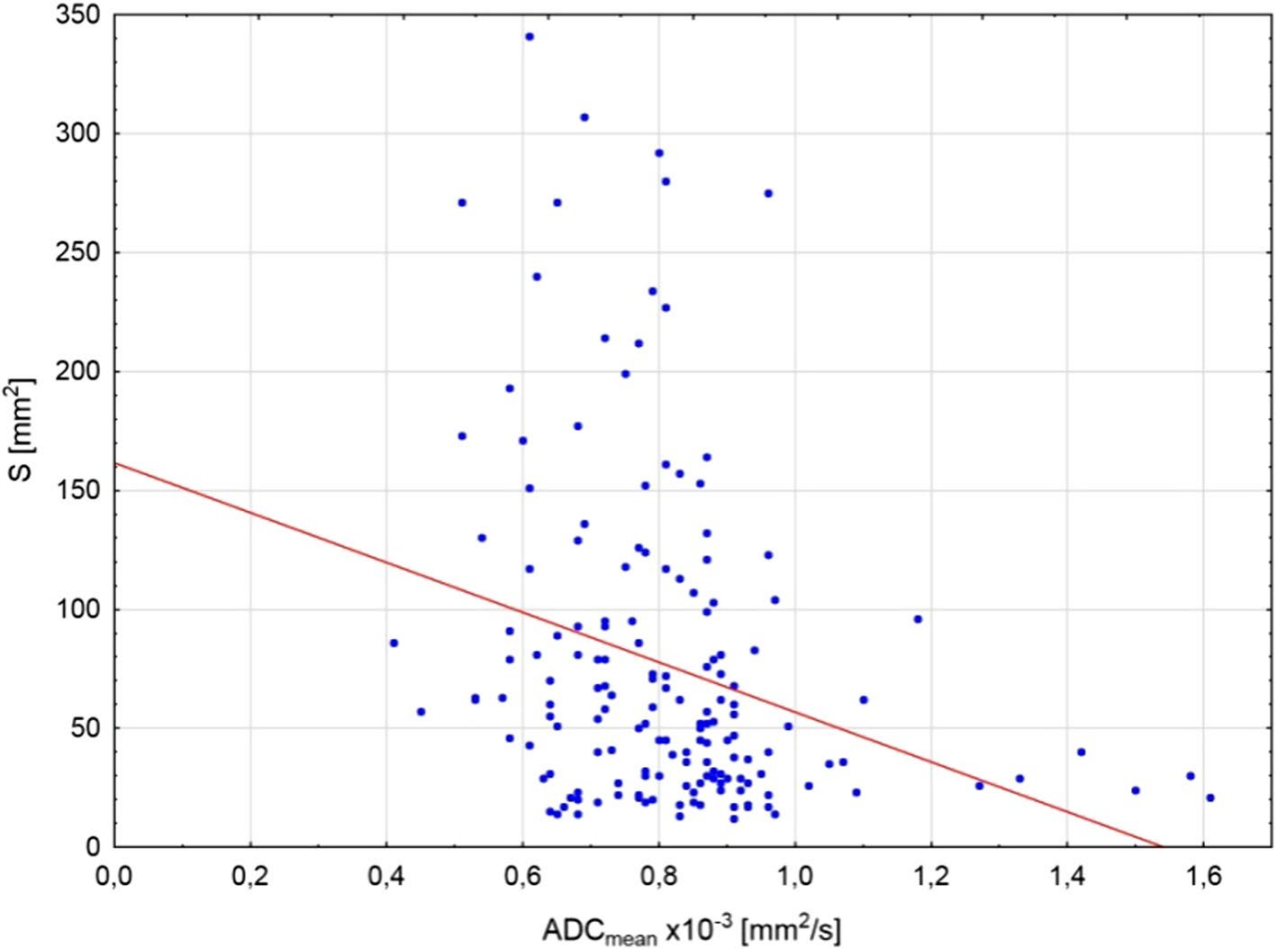
Scatter diagram with regression line occurs lymph node area (*S*) and ADC_mean_ value dependence

Nevertheless, 33 regions with restricted diffusion were not recognized in 18F-FDG PET/CT. These regions were significantly smaller (35.2, SD = 30.9 mm^2^) than localizations visible in both techniques (86.2 SD = 70.3 mm^2^) (*p* = 0.0001). There was a significant negative relationship between the lymph node area and the ADC value, which is confirmed by the Spearman’s rank correlation coefficient (*R*^2^ = − 0.321, *p* < 0.001) (Fig. 3). Detailed information concerning occupied regions together with their SUV and ADC values are presented in Table 2.

**Table 2:** The counts of the identified areas in the two methods of PET/CT and DWIBS, including measured coefficients of SUV and ADC, respectively

Localization/region	DWIBS	ADC × 10^−3^ (mm^2^/s) (SD)	PET/CT	SUV (unitless) (SD)
Cervical	37	0.74 (0.14)	30	10.01 (5.49)
Palatine tonsil	22	0.78 (0.14)	22	7.21 (2.56)
Axilla	20	0.82 (0.09)	12	6.62 (2.64)
Supraclavicular	18	0.81 (0.21)	15	9.64 (4.39)
Subclavicular	18	0.83 (0.26)	15	8.27 (4.15)
Mediastinum	16	1.00 (0.27)	14	10.14 (4.00)
Submandibular	13	0.84 (0.16)	7	6.12 (6.23)
Paratracheal	7	0.86 (0.04)	7	11.54 (5.20)
Paraaortic	6	0.74 (0.17)	6	10.65 (4.14)
Maxillary	4	0.79 (0.04)	2	3.18 (0.47)
Parotid	2	0.86 (0.04)	2	3.46 (0.07)
Visceral	2	0.90 (0.09)	1	13.07
Inguinal	2	0.80 (0.11)	1	1.95
Total	167		134	

Conventional staging procedures were performed as clinically indicated and as recommended by current oncologic protocols. According to the Ann Arbor staging system using 18F-FDG PET/CT and bone marrow biopsy, the disease was stage II (type A, E) in eight patients, in remaining: three patients, stages IIIB, IA and IVA were classified. Classification performed based on WB MRI confirmed results of 18F-FDG PET/CT in nine patients; while in remaining two, it resulted in overstaging.

## Discussion

We showed that the WB MRI and DWIBS techniques allow to recognize FDG-avid lymph nodes and have excellent agreement with the 18F-FDG PET/CT. So far, only two studies (Punwani et al. [41] and Kwee et al. [43]) showed similar results for children. However, what is unique about our study is the narrow group of patients: children with the initial diagnosis of particular type of lymphoma (Hodgkin’s). We assumed that the different types of lymphoma might vary in types of DWIBS findings. Thus, to make the comparison with 18F-FDG PET/CT more reliable, we focused on a homorganic group of great importance. This importance is due to the young age, resulting in an increased risk of future complications due to diagnostics (e.g., radiation) and treatment.

Presented research assessed the WB MRI capabilities to recognize and to perform grading in patients with Hodgkin’s disease. We showed that the WB MR and DWIBS are able not only to confirm the 18F-FDG PET/CT findings but also to inspect more of the lesions than hybrid PET/CT. Based on our research, it can be hypothesized that significant correlation between ADC_mean_ and SUV_max_ indicates that DWIBS may allow to quantitatively assess the disease aggravation. However, due to only fair level of correlation between SUV and ADC, further studies will need to confirm this assumption. At the same time, ADC_mean_ and SUV_max_ were considered as the most reliable, due to the relationship between these metrics, that has been clarified and positively evaluated by some researchers in other lymphoma subtypes [29, 41, 43, 44].

A comparison of our results with the other researches is difficult because there is no other study on comparable Hodgkin’s lymphoma cohort as other studies differ in the applied b parameters, the age of included individuals and the types of examined neoplasms [26, 45]. The meta-analysis by Regacini et al. analyzing cases reports between 2010 and 2013 demonstrated the high specificity of both methods in the primary staging of lymphomas (59–100% of lymph nodes diagnosed with 18F-FDG PET/CT were confirmed with the WB MRI). In addition, it showed the compatibility of these techniques in over 90% [46]. Furthermore, in region-specific analysis, our research confirms the high compliance of coverage of areas in both methods, which is not as good as shown by Albano et al. and Ferrari et al. but occurs greater than in van Ufford et al. paper [47–49]. One of the causes of discrepancies might be a heterogeneous composition of patients groups included in cited studies.

On the other hand, Mosavi et al. [29] reported a lack of compliance between 18F-FDG PET/CT and the WB MRI in the Hodgkin’s lymphoma patients. This lack of compliance may be due to the selection of another age group (17–78 years). There is also no systematic information on whether patients were newly diagnosed. This fact is important because children’s lymphomas are characterized by a specific course and symptoms, unlike adults ones [4]. The last meta-analysis [50], which reported lack of significant relationship between ADC and SUV parameter, explains the variances throughout the biology of different neoplasms [50].

Disparities between DWIBS and 18F-FDG PET/CT are clinically important because they may affect staging of the disease. In our research, 19.76% of areas were diagnosed only by DWIBS which resulted in overstaging in 2 out of 11 patients. In contrast, on Gu et al. [51], overstaging concerns 11.8% of patients; whereas in the research of Abdulqadhr et al. [52] it is only 9.7%.

Furthermore, Stéphane et al. [25] showed that there might be no over-interpretation of DWIBS and that 100% classification coverage in both methods is possible. It should be noticed that in both of these two overstaged cases, additional locations of affected lymph nodes were recognized below the level of the diaphragm. Thus, observed discrepancies were not due to the differences in patient positioning (in the MRI study, patients were placed with hands along the body, while in the 18F-FDG PET/CT, hands were placed behind). Interestingly, pathomorphological evaluation confirmed the nodular lymphocyte-predominant Hodgkin’s lymphoma type in both patients. In this subtype, lymph nodes are densely packed with lymphocytes; thus, diffusion might be profoundly imparted [53]. It may indicate that DWIBS might be a better method for diagnosing patients with this subtype of lymphoma. Nevertheless, further studies including a larger group of patients are required.

Another reason for overstaging may consider the size of analyzed nodules. Overstaging is a consequence of additional lymph nodules recognized in MRI. Additional lymph nodules were significantly smaller than those recognized in both techniques. From the analytic point of view, these additional findings might be treated as the false-positive ones. However, morphological scans and initial follow-up (data not presented) indicate that they were affected. Thus, it rather supports the theory that WB MRI has higher accuracy than 18F-FDG PET/CT.

Finally, discrepancies between DWIBS and 18F-FDG PET/CT might be caused by a lack of ADC diagnostic standards. There are no ADC criteria for differentiating small lymph nodes with low ADC from the same sized, healthy nodes (with no significant diffusion restriction) [19]. On the other hand, the ADC is the only technique that allows for evaluating signal intensity criteria for discriminating between lymphomatous and normal lymph nodes. Nevertheless, it is only reported that ADC is lowest in lymphomatous nodes and highest in healthy ones [31].

Taking into account all the above, the critical task for making WB MRI with DWIBS technique a reliable diagnostic method is to determine diagnostic references of ADC [25, 54]. Our analysis showed that in all affected lymph nodes, the apparent diffusion coefficient was lower than 1 × 10^−3^ mm^2^/s. It is only the simple fact, without further conclusions considering this value as a cut point on the ADC scale. Additional researches on a large cohort are required to determine referential values. Moreover, the technique of ADC measurement should be unified. Measurement of an ADC value is somewhat challenging and may vary between different centers because of the different ADC measurement parameters. The ADC measurement can also be negatively affected by tissue heterogeneity and respiratory artifacts [55]. According to Kwee and Takahara, free breathing is referred to as the “driving force” in the DWIBS sequence [56]. Nevertheless, they pointed out that diaphragm movement can disturb the visualization of small-sized lymph nodes placed around the spleen or liver [44]. In a more recent study, Stone et al. [57] designated that mean ADC values are not much affected by breathing, but there is a significant increase in the spread of standard deviation of ADC values, what may be attributed to blurring effects. The solution may be to use *Navigator-based triggering* or other tracking systems [58].

In summary, presented results indicated that the mean ADC can potentially discriminate between healthy and lymphomatous lymph nodes. Also, DWIBS could be a useful, non-invasive and radiation-free functional imaging method for visualizing lymphomatous lesions in children. Furthermore, ADC can be used for a quantitative evaluation of process aggravation [59]. Thus, ADC might become a biomarker of the Hodgkin’s lymphoma course and a diagnostic tool of choice instead of single CT examination [20, 30]. Thus, the use of DWIBS instead of single CT may initiate changes in oncologic trials in the future, especially in pediatric patients.

### Limitations

The first limitation of present study is lack of comparison between ADC and SUV in extranodal regions. Organs which can be affected by lymphoma are liver, uterus, skeletal system and bone marrow. Analysis of extranodal regions is mandatory to properly classify lymphoma according to Ann Arbor system. Despite the fact that our group of patients consisted of those who were mainly classified in the second stage of disease (no extranodal regions affected) and that the bone marrow involvement does not frequently appear among Hodgkin lymphoma patients, measuring the ADC parameter in all extranodal structures has a diagnostic potential [60] and would be performed in our future study. So far, PET examination was the one which affirmed bone marrow involvement with high accuracy; however, this standard could be changed in the future in favor of diffusion-weighted imaging precision [61–64].

Secondly, despite the spatial resolution difference between WB MRI and 18F-FDG PET/CT, no direct calculation to assess these differences was performed. The role of the PET/CT examination in oncological trials is widely accepted; however, there were first reports which verified that MRI has a better ability to differentiate smaller areas than 18F-FDG PET/CT [65, 66]. We demonstrated two parameters: SUV and ADC, which correlate with spatial resolution in 18F-FDG PET/CT and MRI studies, respectively. However, quantitative assessment of the resolution would be our goal in the follow-up.

The next potential limitation comes from the presumption that the selected *b* values of 0 and 800 are optimal for DWIBS examination in children. The ADC decrease is inversely proportional to the *b* value [67]. Thus, there is no optimal choice of *b* values in WB DWIBS. According to our research, the choice was made by an experienced radiologist based on the observations and general knowledge [39, 41]. We are also familiar with the paper by Koc and Erbay, where they listed the most valuable *b* values [68]. Thus, there is a papers where b0 and b1000 or combination of three *b* values are used. However, we decided to use b800 and b0 pair to reach optimal SNR and to gain a study time [69]. Other researches confirmed efficacy of b800 to recognize all lymph nodes with restricted diffusion [62, 66, 69].

Another limitation concerns lack of 18F-FDG PET/CT scanner EARL-accreditation. It enables a comparison of quantitative results with other centers. Nevertheless, it does not bias our findings of the correlation between PET/CT and DWIBS.

Finally, there is a potential bias in our analysis, as we evaluated correlation between ADC_mean_ vs SUV_max_ in large number of lymph nodes, but derived only from 11 patients. Thus, outlining results in a single patent might be multiplied when evaluating all lymph nodes together. To reduce this risk, we controlled for outliers and observed homogenous trends in all patients.

## Conclusion

Our study showed that the WB MRI with DWIBS technique is useful in identifying and staging affected lymph nodes in the pretreatment group of newly diagnosed children with Hodgkin’s lymphoma. This method is also superior to 18F-FDG PET/CT in recognizing small lesions. WB MRI examination, firstly, allows to obtain high-resolution diagnostic images. Secondly, it allows to perform evaluation without contrast and radiation in a relatively short time. It may shed light on future perspective to avoid additional CT scan which is now attributed to standard oncologic protocols. Thus, our initial research may give a foundation to create a universal diagnostic model dedicated to diagnosing Hodgkin’s lymphoma in peadiatric patients.
